# The effect of NF-kB and MAPK mediated Proinflammatory microenvironment on renal aging and amyloid deposition in elder rats

**DOI:** 10.1038/s41598-025-14559-y

**Published:** 2025-08-18

**Authors:** Mete Keçeci, Cenk Murat Özer, Esra Babaoğlu, Furkan Bodur, Ali Can Önal, Ayşe Zeynep Yılmazer Kayatekin, Osman Cengil, Ali Serdar Karataş

**Affiliations:** 1 Faculty of Medicine, Department of Histology and Embryology, Zonguldak Bülent Ecevit University, Zonguldak, Turkey; 2 Faculty of Medicine, Department of Anatomy, Tekirdağ Namık Kemal University, Tekirdağ, Turkey; 3 Faculty of Medicine, Department of Anatomy, Zonguldak Bülent Ecevit University, Kozlu/Zonguldak, 67600 Turkey; 4 Faculty of Medicine, Department of Pathology, Zonguldak Bülent Ecevit University, Kozlu/Zonguldak,, Turkey

**Keywords:** Aging, Inflammation, NF-κB, MAPK, TNF-α, IL-6, Occludin, GRP78, Kidney, Amyloidosis, Cell biology, Anatomy, Health care

## Abstract

Low-grade inflammation associated with the aging process exerts functional and morphological effects on multiple organs. The kidney is an organ that is severely affected by the aging process. Our study aimed to determine the relationship between histopathological and immunohistochemical changes and age-related amyloid accumulation in rat kidney tissue. *Wistar albino* rats were divided into two groups: young adult and elder (*n* = 8, 24 and 104 weeks old, respectively). H&E, PAS, and Congo red staining and immunohistochemical staining for TNF-α, IL-6, occludin, GRP78, and mitogen-activated protein kinase (MAPK) were performed on the kidney tissues of the subjects. A significant difference was found between the groups in terms of glomerulus diameter/Bowman’s capsule diameter, percentage of sclerotic glomeruli, epithelial desquamation, loss of brush border, casts, tubular dilatation, cellular debris, and the presence of apoptotic cells. TNF-α, IL-6, GRP78, and MAPK protein levels were shown to increase significantly in aged rat kidneys, whereas occludin levels decreased significantly. No amyloid accumulation was found in the kidney, heart, skin, and small intestine tissues of either group. The results of our study suggest that the proinflammatory microenvironment required for AA amyloid deposition in systemic amyloidosis induced by low-grade inflammation in old age cannot be generated by the action of NF-κB, MAPK, and MAPK-activated JNK and AP-1 transcription factors alone.

**Supplementary Information:**

The online version contains supplementary material available at 10.1038/s41598-025-14559-y.

## Introduction

Aging is a natural, biologically irreversible process in which organisms gradually lose their functions at the cellular and molecular level over time. This process is shaped by the interaction of many variables such as genetic factors, environmental factors and lifestyle. With aging, deteriorations occur in various physiological systems, which pave the way for the development of common health problems such as cardiovascular diseases, neurodegenerative diseases, diabetes mellitus, osteoarthritis and renal dysfunction. Chronic inflammation and oxidative stress are among the basic pathophysiological mechanisms of aging-related diseases, and controlling these processes plays a critical role in the prevention of age-related health problems^1,2^. Concurrently, aging has emerged as a significant public health concern, affecting both individuals and society at large. Age-related health issues can lead to a decline in quality of life. Furthermore, the financial burden of managing these conditions contributes to a growing social and economic strain^3^.

The constant activation of the inflammatory response and the increase in proinflammatory cytokines play a crucial role in the pathogenesis of aging-related diseases. During this process, signaling pathways such as NF-κB and MAPK are activated, sustaining inflammation. Additionally, increased oxidative stress accelerates cellular aging, leading to mitochondrial dysfunction, protein homeostasis disruption, and impaired autophagy^4^ These mechanisms contribute to structural and functional disorders in various organs, particularly in kidney tissue, during aging. Today, various strategies—such as pharmacological agents with anti-inflammatory and antioxidant properties, nutritional adjustments, and exercise—are employed to prevent and treat aging-related diseases. These approaches aim to suppress the inflammatory response, reduce oxidative stress levels, and support cellular repair processes^5^.

The aging process affects all tissues and organs, but the kidney is among the organs that undergo the most significant morphological and functional changes^6^. As a consequence of aging, a phenomenon known as ‘low-grade inflammation’ emerges, characterized by the continuous production of inflammatory mediators at low levels^7^. In the kidneys, low-grade inflammation triggered by aging can disrupt repair processes after damage and lead to organ damage. Consequently, fibrosis in kidney tissue, thickening of glomerular and tubular basement membranes, focal segmental glomerulosclerosis, tubular epithelial damage, deterioration in mitochondrial functions and disruption of the autophagy pathway used by proximal tubule epithelial cells to eliminate abnormal organelles, especially mitochondria, may occur^8–11^. The process of inflammation is not only responsible for observable morphological alterations in the kidney, but also for deviations from normal at the molecular level. Xu et al. showed that hyperoxia-induced increase in TNF-α and IL-6 expressions in kidney tissue in neonatal rats decreased occludin expressions^12^. Han et al. showed that there was a negative correlation between the increase in TNF-α, IL-1 and IL-6 levels and renal tubular occludin expression in rats^13^.

Amyloidosis refers to a group of pathological conditions caused by the accumulation of aberrantly folded autologous proteins in the extracellular space. This process is closely linked to endoplasmic reticulum (ER) stress, and the kidney is among the organs most susceptible to amyloidosis^14^. Kamada et al. demonstrated that lysozymes containing point mutations I56T, F57I, W64R, and D67H in the HEK293 cell line accumulated within the cells, increasing the levels of ER stress markers, including Glucose Regulated Protein 78 (GRP78) and IRE1α^15^. Serum amyloid A (SAA) is an acute-phase protein synthesized in the liver during the acute phase of inflammation. Its serum level can be elevated to a greater extent than C-reactive protein, even with minimal levels of inflammatory stimuli, particularly IL-1 and IL-6. The proteolytic cleavage of SAA results in the formation of AA amyloid^16^. There are also studies linking the low-grade inflammation that occurs with aging to many pathologies, including neurodegenerative diseases, ovarian dysfunction, intestinal barrier disorders, renal dysfunction, and amyloidosis^17–20^. The accumulation of amyloid fibrils plays an important role in the pathogenesis of fatal age-related disorders such as degenerative central nervous system diseases and senile systemic amyloidosis in the elderly. In addition, senile amyloidosis can have a cumulative effect on organ function deterioration. For example, the deterioration of renal function due to amyloid deposition can lead to the rapid decline of myocardial function, which is already compromised in the elderly^21,22^. This fact underscores the importance of experimental studies that examine both aging and amyloidosis together.

In our study, we specifically investigated the relationship between NF-κB and MAPK-mediated proinflammatory microenvironment and amyloid deposition in aged rat kidneys. In this context, our study aimed to determine the histopathological, histomorphometric, and immunohistochemical changes that occur in the kidney tissue of *Wistar albino* rats due to aging, and to assess whether low-grade inflammation associated with aging is a sufficient cause of amyloid deposition. This study provides important findings to understand the effects of aging-related inflammatory processes on amyloid deposition. It may contribute to the identification of new therapeutic targets to protect kidney health in advanced ages.

## Methods

### Animals and ethics

In our study, 24-week-old young adult female *Wistar albino* rats weighing 250–300 g (young group) and 104-week-old female Wistar albino rats weighing 350–400 g (elder group), produced at the Zonguldak Bulent Ecevit University Animal Care and Research Unit (Zonguldak, Turkey), were used to form two groups of eight subjects each. A total of 16 rats were included in the study. During the experiment, suitable environmental conditions for animal care (20 ± 1 °C room temperature, 60 ± 10% humidity, and 12/12 hour light/dark cycle) were provided and subjects were allowed free access to food and water. Prior to conducting this study, approval was obtained from the Bülent Ecevit University Institutional Animal Ethics Committee (Zonguldak, Turkey; 2023-15-07/09). All procedures performed throughout the experiment were carried out in accordance with the Guide for the Care and Use of Laboratory Animals, published by the US Public Health Service. All sections of this report adhere to the ARRIVE Guidelines for reporting animal research^23^. A completed ARRIVE guidelines checklist is included in Checklist S1.

The sample sizes in the study were calculated using G*Power 3.1 software, with a significance level (α) of 0.05, a power (1 - β) of 0.80, and an effect size (r) of 0.50^24,25^.

### Histopathological and histomorphometric evaluations

**Fig. 1:**
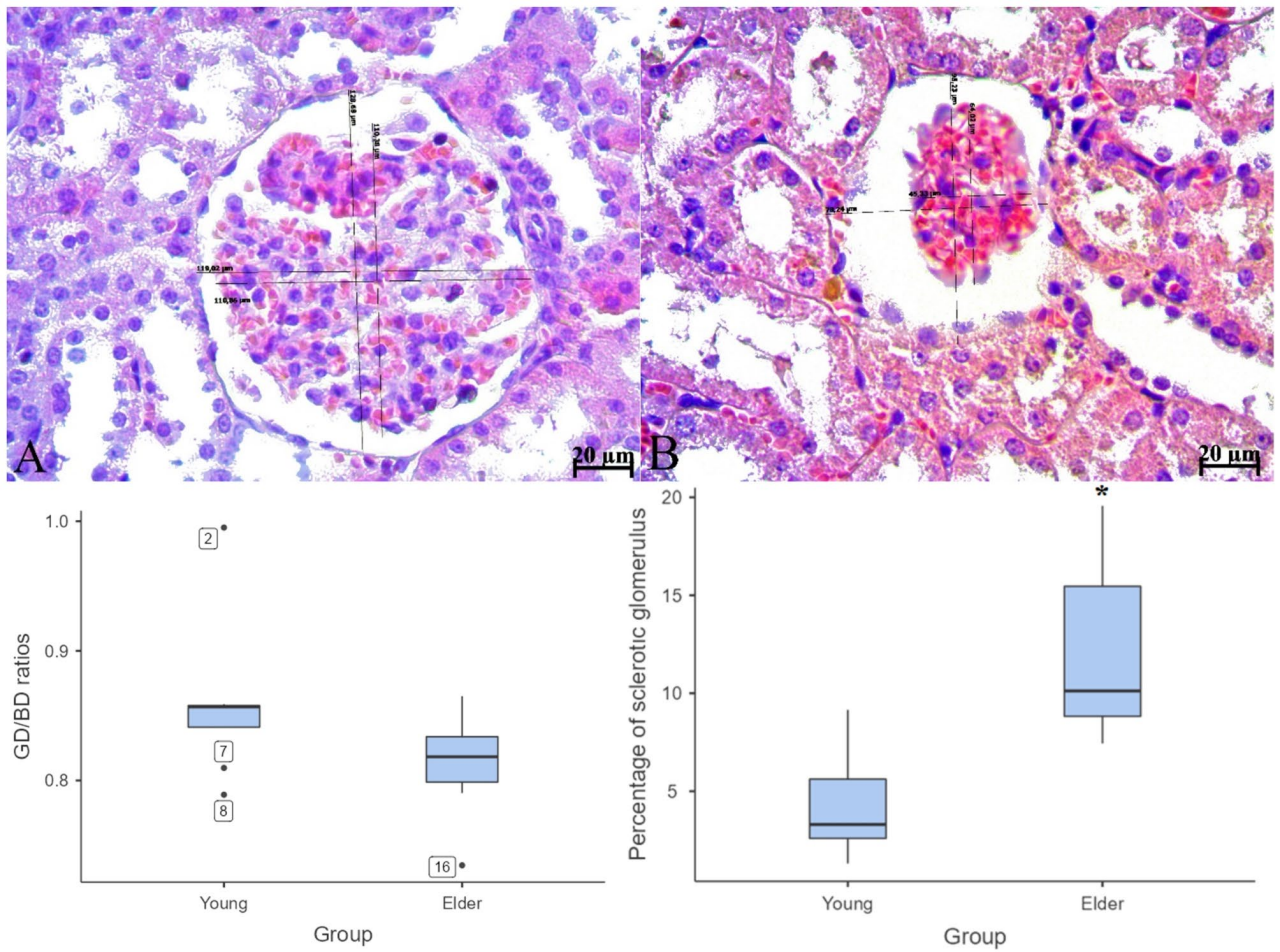
Diameter measurement method of Bowman’s capsule and glomerulus (A; young, B; elder groups) and results of statistical analysis of GD/BD ratios and percentage of sclerotıc glomerulus calculated for young and elder subjects. Scale bar; 20 μm. Data are presented as median (min-max). *p<0.01.

At the conclusion of the experiment, the subjects were anaesthetised with ketamine (90 mg/kg) and xylazine (10 mg/kg) and then euthanised by exsanguination via the abdominal aorta using a 21 G syringe. In order to reveal histopathological and histomorphometric changes occurring in the rat kidney due to aging, kidney tissues were removed and divided into two parts with a coronal incision passing through the extremities superior, renal hilus and extremities inferior. Following fixation with 10% formalin, paraffin blocks were obtained by applying routine tissue processing procedures to the tissues. 5 μm thick sections were taken from the same tissues and stained with hematoxylin-eosin (H&E), periodic acid-schiff (PAS) and Masson trichrome staining methods. In order to demonstrate the expansion or contraction of the renal glomeruli, the measurement method used by Tarladacalisir et al. was used with modification^26^. In H&E stained tissues, glomerulus and Bowman capsule diameters were measured in 20 sections for each slide and glomeruli were counted in the widest section obtained for each kidney tissue. The widest diameter of the Bowman capsule and glomerulus was measured first. Subsequently, the second diameter of the Bowman capsule and glomerulus was measured perpendicular to the midpoint of the first measured diameter, and the arithmetic mean was calculated. Using these values, the ratio of the glomerulus diameter to the Bowman capsule diameter was determined (Fig. 1). To calculate the percentage of sclerotic glomeruli, random glomeruli were evaluated in 20 fields. Glomeruli were considered sclerotic if they exhibited either shrinkage of the glomerular tuft, resulting in an expanded Bowman’s space, or expansion of the glomerular tuft due to increased mesangial connective tissue, leading to the obliteration of Bowman’s space. The percentage of sclerotic glomeruli for each evaluated area was calculated using the following formula: (Number of sclerotic glomeruli observed in the evaluated area/Total number of glomeruli observed in the evaluated area) × 100. All analyses were conducted using the x20 magnification objective of the microscope. Histological changes due to aging were scored by the percentage of tubules showing epithelial desquamation, loss of brush border, casts, tubular dilatation and the presence of debris and apoptotic cells (cells with pyknotic nuclei, increased cytoplasmic eosinophilia, and reduced size were considered apoptotic) as follows: 0, none; 1, ≤ 10%; 2, 11–25%; 3, 26–45%; 4, 46–75%; and 5, > 76%. A minimum of ten fields were evaluated for each slide^27^. Brush border loss was evaluated in slides stained with the PAS method, and other parameters were evaluated in slides stained with H&E. In addition, Masson trichrome staining method was applied to reveal the changes that occur in the connective tissue of the kidneys due to aging. Slides were evaluated and photographed using a light microscope (Carl Zeiss Axio Lab A1, Jena, Germany).

### Assessment of amyloid deposition

To determine whether mere aging makes a difference in terms of amyloid accumulation, Congo red standard staining protocol was applied to 5 μm thick sections taken from kidney, heart, small intestine and skin tissues, and the yellow-green birefringence obtained under polarized light was interpreted as amyloid deposition.

### Immunohistochemical evaluations

For immunohistochemical detection, 3 μm thick sections were obtained from paraffin blocks on positively charged slides. After deparaffinization and immersion, heat-induced epitope retrieval was performed in citrate buffer. Sections were then placed in distilled water and allowed to cool at room temperature for 20 min. After three washes with PBS, sections were treated with 3% hydrogen peroxide for 10 min to block endogenous peroxidase activity. After Ultra V blocking (Thermo Fisher Scientific, Massachusetts, USA) for seven minutes to block non-specific binding sites, sections were treated with anti-TNF-α (PA1-40281, 1:1000, Thermo Fisher Scientific, Massachusetts, USA), anti-IL-6 (NB600-1131, 1:1000, Novus Biologicals, Cantennial, USA), anti-occludin (PA5-105057, 1:1500, Thermo Fisher Scientific, Massachusetts, USA), anti-GRP78 (ab21685, 1:800, Abcam, Cambridge, UK) and anti-p44/42 MAPK (3085R-100, 1:1000, Biovision, Paris, France) primary antibodies (24 h at + 4 °C). After washing with PBS, secondary antibodies (Thermo Fisher Scientific, Massachusetts, USA) and streptavidin-peroxidase (Thermo Fisher Scientific, Massachusetts, USA) were applied for 30 and 10 min respectively at room temperature. The sections were then treated with diaminobenzidine (DAB) chromogen solution (Vector, SK-4100) until the desired staining intensity was achieved under a light microscope and counterstained with haematoxylin. During all incubations, the tissues were kept moist to prevent desiccation and consequent background staining. Sections were closed with entellan and coverslips were examined under a light microscope. A Zeiss Axio Lab. A1 photomicroscope was used. All immunohistochemical analyses were performed according to the protocols recommended by the manufacturer. Histological scoring (H-SCORE) was performed manually using the following criteria to define immunohistochemical results: 0: no staining, 1+: weak but detectable staining, 2+: moderate to strong staining, 3+: strong staining. The H-SCORE value for each section was obtained by multiplying the percentage of stained cells for each density category by its density. Scoring was done under the light microscope at x40 objective magnification on 20 randomly selected fields on each section and mean scores were used for statistical analysis. H-score = ∑i i xPi, i; density score, Pi; cell percentage^28^. Additionally, a ‘secondary antibody only control’ staining was carried out to confirm that the secondary antibody did not bind non-specifically to cellular components, ensuring the specificity of the staining results. For this purpose, kidney tissues were treated with antibody diluent alone during the primary antibody incubation stage. The subsequent steps of immunohistochemical staining were performed as described above.

All tissue processing and staining protocols were performed as described by Suvarna et al.^29^.

### Statistical analysis

The statistical analysis of the data was conducted using the Jamovi 2.3.21 package program. The normality of the continuous variables was evaluated using the Shapiro-Wilk test. The descriptive statistics of the quantitative variables are presented with the mean, standard deviation, median, minimum, and maximum values. The Mann-Whitney U test was employed to compare the quantitative independent variables between two groups. A value of *p* < 0.01 was considered significant.

## Results

### Histopathological findings

**Fig. 2:**
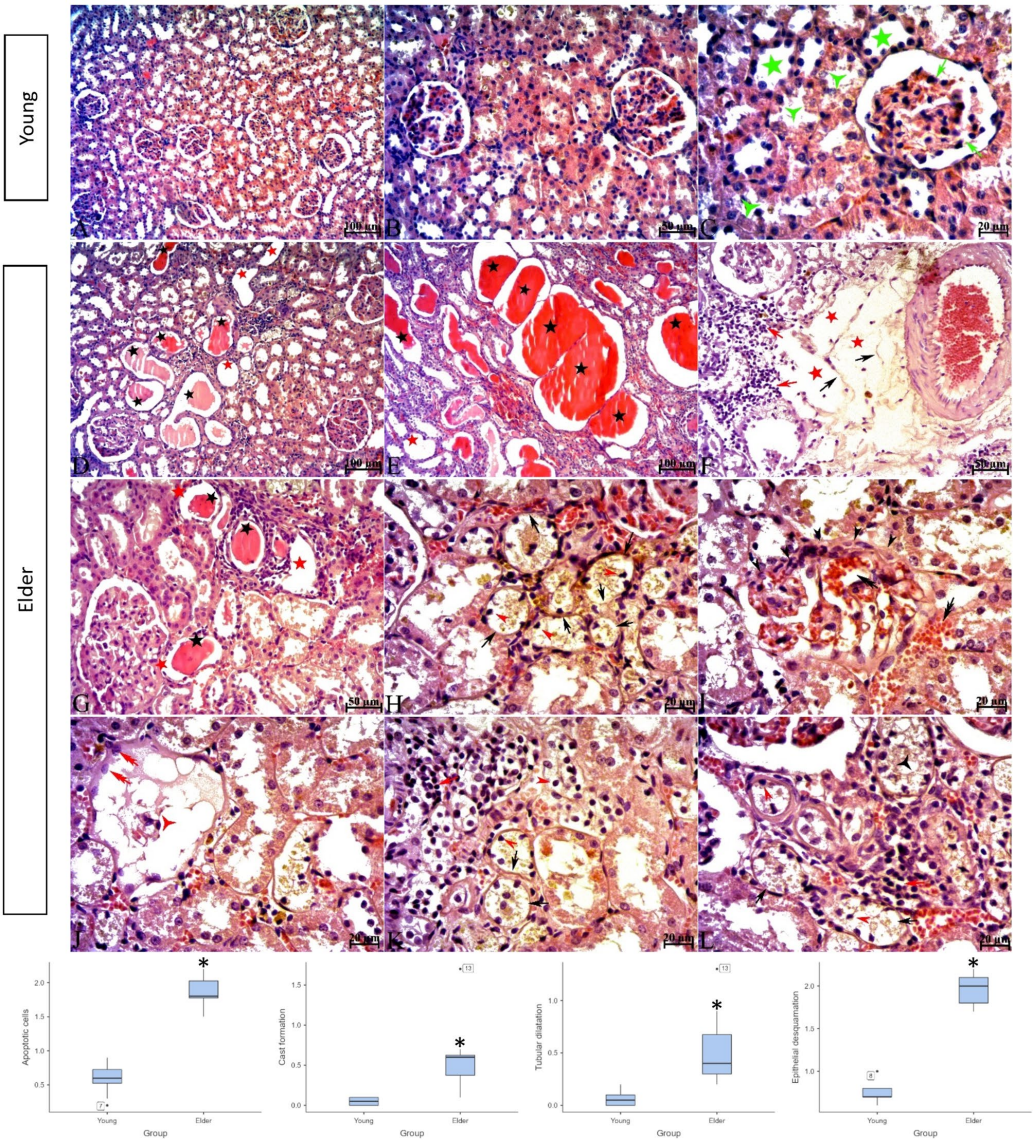
H&E staining images and histopathological scoring results of kidney tissues of young and elder groups. A, B, C; Young, D, E, F, G, H, I, J, K, L; Elder. Normal looking glomeruli and tubules were observed in young rats. Three-armed green star; normal looking proximal tubule, five-armed green star; normal looking distal tubule, green arrow; normal looking glomerulus. In the elder group cast formation (black star), tubular dilatation (red star), inflammatory cells (red arrow), sclerotic glomeruli (red three-armed star), synechiae and narrowing of Bowmann space (black arrowhead), glomerular tubulization (red double-headed arrow), capillary congestion and dilated glomerular capillaries (black double-headed arrow), pyknotic nucleus (red arrowhead), epithelial desquamation (black arrow), cellular debris (black three-armed star) were observed. Scale bar; A, D, E; 100 μm, B, F, G; 50 μm, C, H, I, J, K, L; 20 μm. *p < 0.01. Data are presented as median (min-max).

**Fig. 3:**
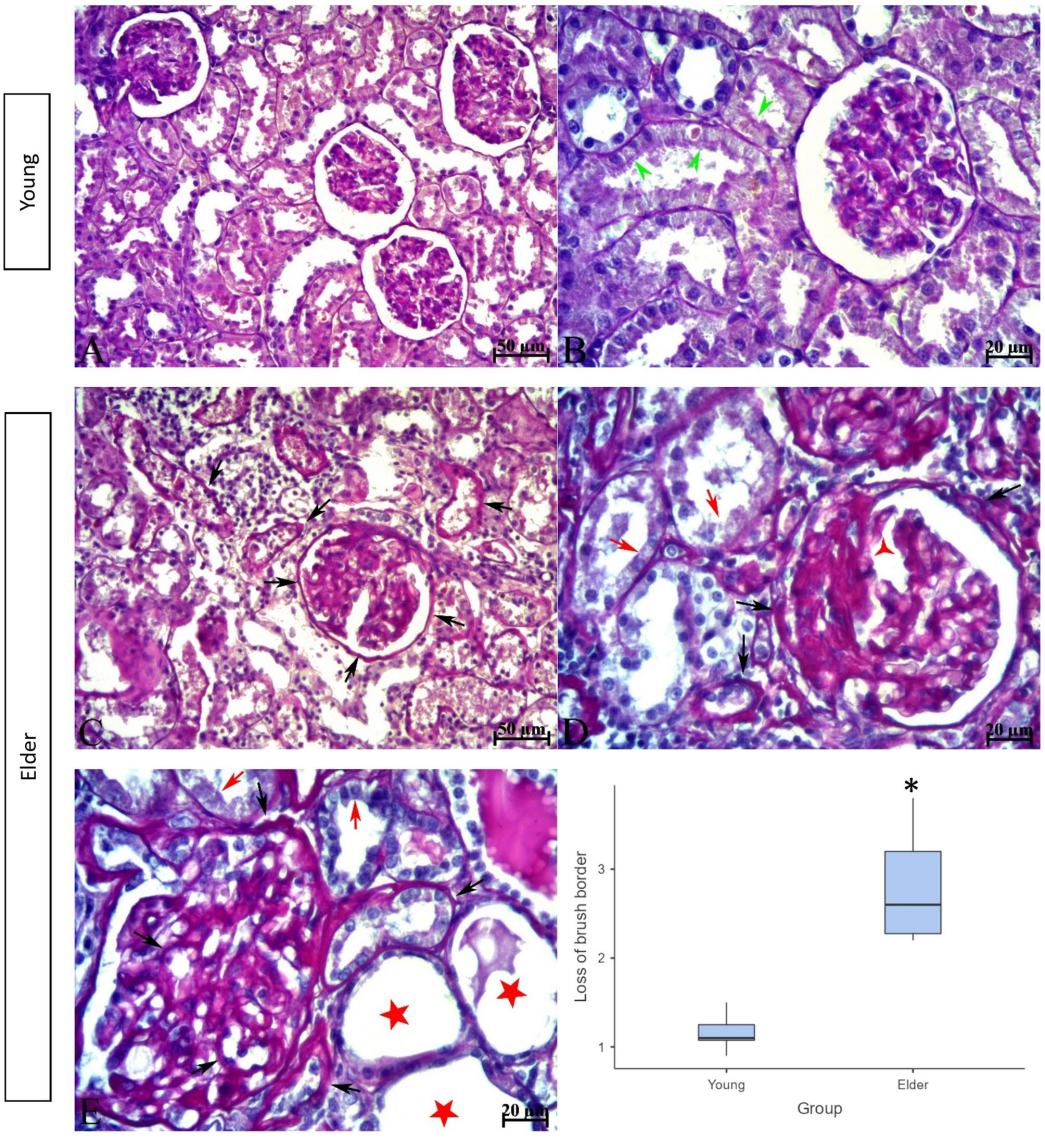
PAS staining images and histopathological scoring result to evaluate the brush border of kidney tissues of young and elder groups. A, B; Young adult, C, D, E; Elder. In the young group, normal-appearing glomerular and tubular basement membranes and a brush border with preserved integrity were observed (green arrowhead). In the elder group basement membrane thickening or irregularity of the basement membrane contour (black arrow), loss of brush border (red arrow) were observed. Tubular dilatation with flattened epithelium (red star), segmental sclerosis in the glomerulus (red three-armed star). Scale bar; A, C; 50 μm, B, D, E; 20 μm. *p < 0.01. Data are presented as median (min-max).

**Fig. 4:**
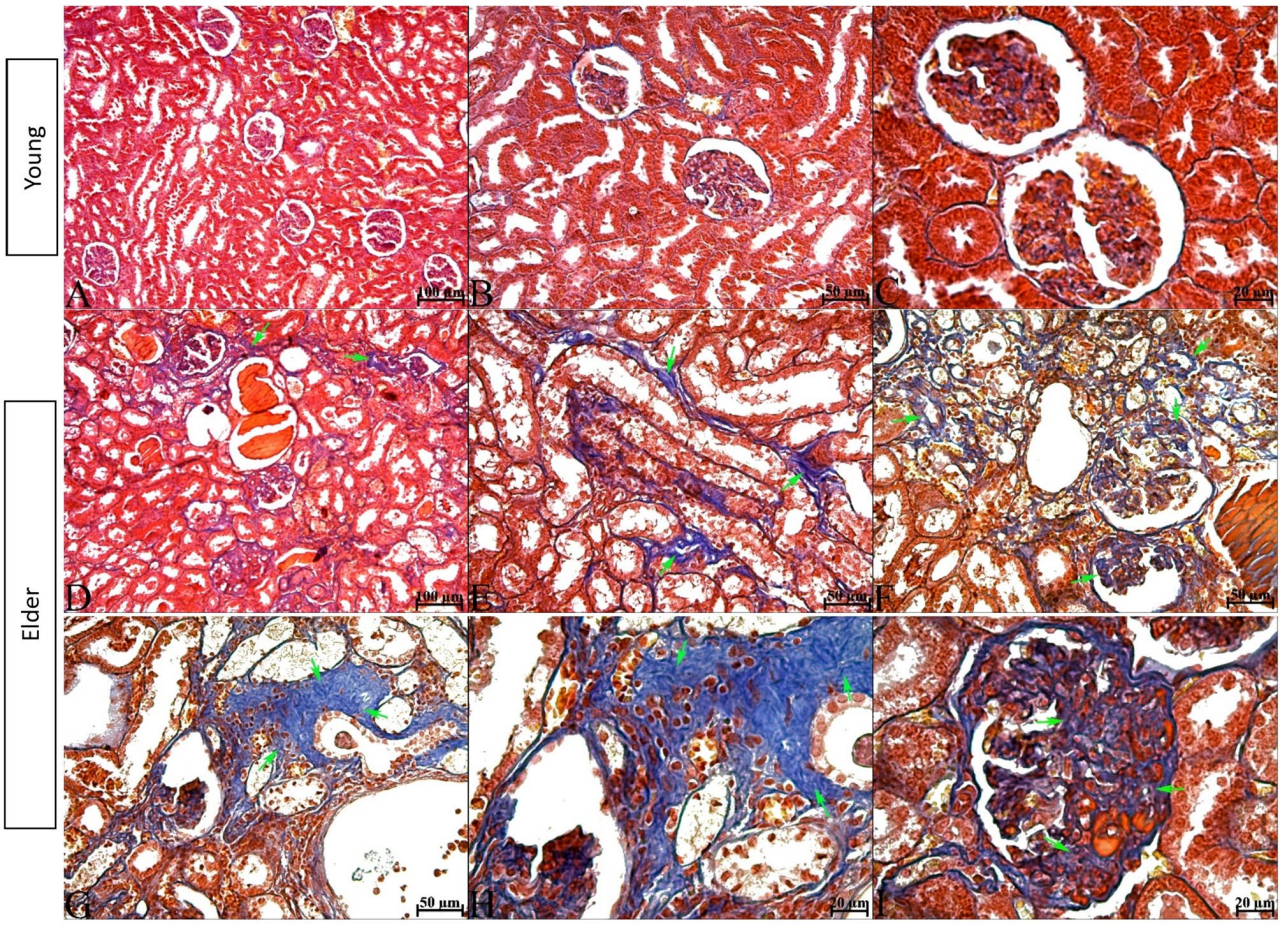
Masson trichrome staining images of kidney tissues of young and elder groups. In the young group, inter-tubular connective tissue and glomerular mesangium appeared normal. In the elder group, blue staining showing collagen fibre accumulation both in the connective tissue between the tubules and in the glomerular mesangium is remarkable (green arrow). A, B, C; young, D, E, F, G, H, I; elder. Scale bar; A, D; 100 μm, B, E, F, G; 50 μm, C, H, I; 20 μm.

H&E-stained kidney tissue sections from subjects in the young group had normal histological architecture. In contrast, interstitial inflammatory cell infiltration, tubular dilatation, manifested by flattened epithelium and enlarged lumens, and widespread protein casts, more prominent within the enlarged tubules, were observed in the kidney tissue of subjects in the elder group. Additionally, there was epithelial desquamation, epithelial cells with pyknotic nuclei, and cellular debris in the tubule lumens. Sclerotic glomeruli were predominantly observed to be consistent with advanced-stage glomerulosclerosis, characterized by glomerular tuft contraction and expansion of Bowman’s space. However, glomeruli consistent with early-stage glomerulosclerosis were also present, distinguished by an enlarged glomerular tuft that narrowed or, in some areas, obliterated Bowman’s space. These glomeruli showed dilation and congestion in the capillary lumen. In a number of sclerotic glomeruli, the epithelium of the outer layer of Bowman’s capsule was observed to undergo a transition from a monolayer of squamous epithelium to a monolayer of cuboidal epithelium (glomerular tubulisation), indicating metaplasia. (Fig. 2). In the evaluation of tissues stained with PAS reaction, brush border irregularity or complete loss on the apical surfaces of proximal tubule epithelial cells and thickening and irregularity of the tubule basement membrane contour in the renal tubules were observed in the elder group. Basement membrane thickening and irregularity of the basement membrane contour were also seen in the outer layer of Bowman’s capsule and glomerular capillary basement membranes (Fig. 3). As a result of histological scoring, which evaluated the presence of debris and apoptotic cells, cast formation, tubular dilatation, epithelial desquamation, and irregularity or complete loss of the brush border, a statistically significant difference was found between the groups for all criteria (The median (min-max) values ​​for all parameters for the young and elder groups were; Apoptotic cells; 0.60 (0.20–0.90) and 1.80 (1.50–2.20), Cast formation; 0.05 (0.00-0.10) and 0.60(0.10–1.70), Tubular dilatation; 0.05 (0.00-0.20) and 0.40 (0.20–1.30), Epithelial desquamation; 0.70 (0.60-1.00) and 2.00 (1.70–2.20), Loss of brush border; 1.10 (0.90–1.50) and 2.60 (2.20–3.80), *p* < 0.01 for all criteria) (Figs. 2 and 3). Masson trichrome staining of kidney sections from subjects in the young group revealed that the renal interstitium, located between the tubules and the glomerular mesangium, had a normal appearance. In contrast, in the elder group, an increase in the mesangium was observed in some glomeruli. The Bowman’s spaces associated with these glomeruli were narrowed. Additionally, a marked increase in the interstitial connective tissue was evident, particularly around tubules exhibiting epithelial degeneration and dilatation (Fig. 4).

### Histomorphometric findings

No statistically significant difference was found when comparing the GD/BD ratio between groups (*p* = 0.130). However, the mean GD/BD ratio was lower in the elder group (the GD/BD ratio was 0.86 ± 0.06 and 0.81 ± 0.04 in the young and elder groups respectively). When the percentages of sclerotic glomeruli were compared, it was observed that the percentage of sclerotic glomeruli in the elder group was significantly higher than in the young group (*p* < 0.01). The percentage of sclerotic glomeruli in the young and elder groups was 4.31 ± 2.65 and 12.03 ± 4.35 respectively (Fig. 1).

### Amyloid deposition

**Fig. 5:**
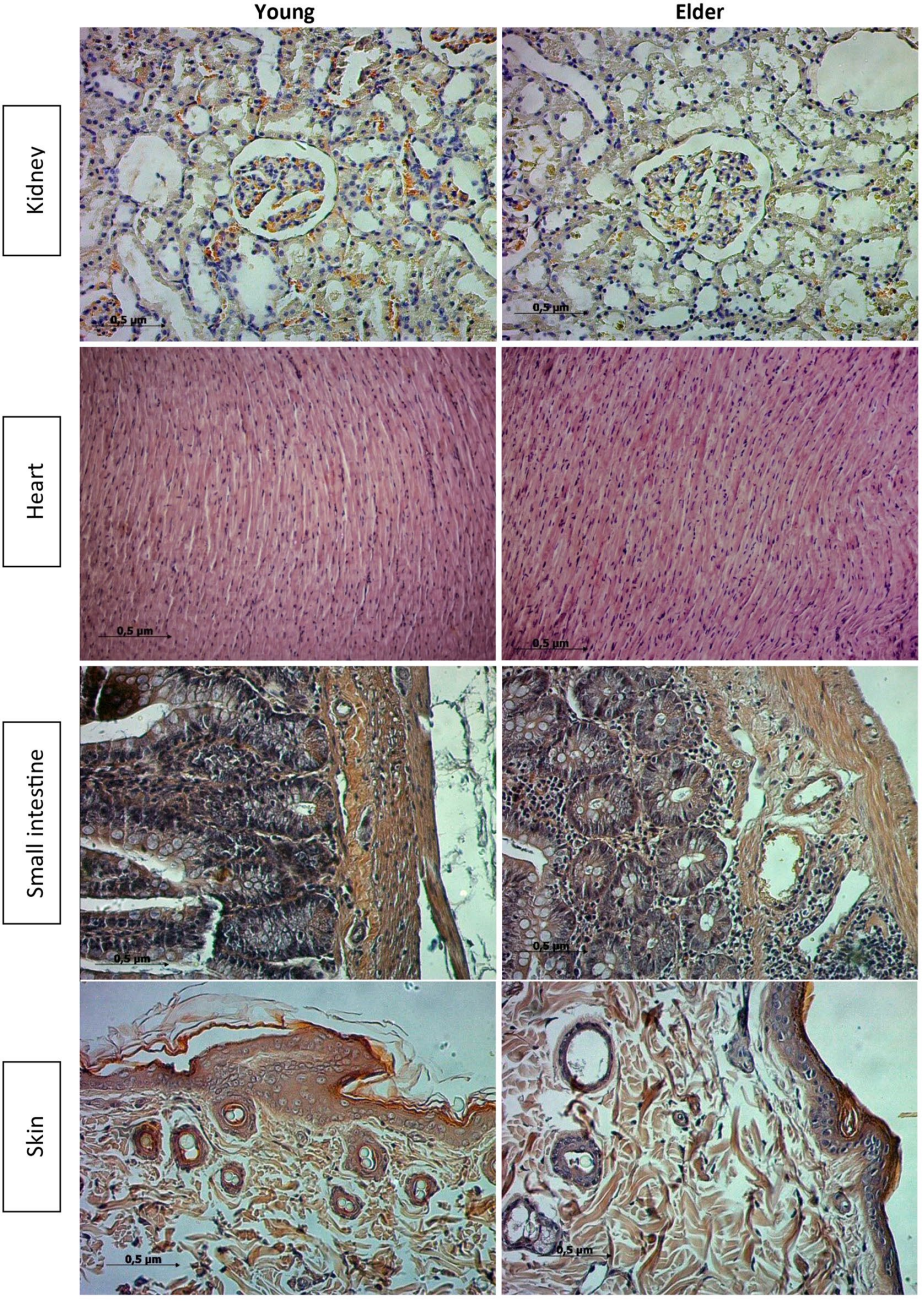
Polarization microscope images of kidney, heart, small intestine and skin tissues stained with Congo red dye. No yellow-green birefringence suggestive of amyloid deposition was observed on polarisation microscopy in either young or elder rats. Magnification 200x.

The results of Congo red staining, which was performed to demonstrate the presence of amyloid accumulation in the kidney, heart, skin, and small intestine tissues of the subjects, indicated that amyloid accumulation was not observed in either the young or the elder group (Fig. 5).

### Immunohistochemical findings

**Fig. 6:**
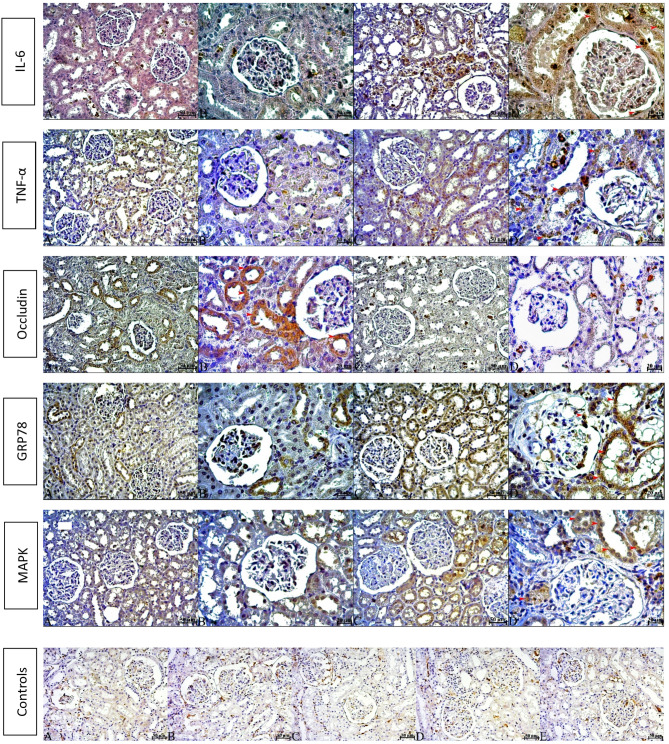
Immunohistochemical staining results for IL-6, TNF-α, Occludin, GRP78 and MAPK proteins of kidney tissues of the groups. The red arrowhead indicates a positive reaction. A, B; young, C, D; elder. Scale bar; A, C; 50 μm, B, D; 20 μm. Secondary antibody only control A; IL-6, B; TNF- α, C; Occludin, D; GRP78, E; MAPK. Scale bar; 50 μm.

The immunohistochemical staining revealed that the protein levels of IL-6, TNF-α, GRP78, and p44/42 MAPK were significantly higher in the elder group than in the young group (*p* < 0.01 for each). A comparison of occludin protein levels between the two groups revealed a statistically significant reduction in tissue occludin levels in the elder group relative to the young group (*p* < 0.01). The median (min-max) values ​​for all antibodies in the young and old groups were; IL-6; 101 (76–127) and 174 (142–184), TNF-α; 36.00 (31–45) and 145.50 (118–167), GRP78; 56.5 (23–90) and 203 (103–260), p44/42 MAPK; 48.5 (38–81) and 185 (133–239), occludin; 187.25 (149–257) and 106.08 (48.3–141). In all immunohistochemical stainings performed, either no staining or very weak staining was observed in the glomeruli. Reactions were especially evident in the tubules. (Fig. 6; Table 1).

**Table 1:** Statistical analysis of h-score values ​​obtained from immunohistochemical staining to show the levels of IL-6, TNF-α, occluding, GRP78, and MAPK proteins in the kidney tissues of young and old subjects. Values ​​are given as median (min.-max.). p<0.01 significant when compared with the young group.

	Young (*n* = 8)	Elder (*n* = 8)	*p*
IL-6	101 (76–127)	174 (142–184)	< 0.001
Occludin	187.25 (149–257)	106.08 (48.3–141)	< 0.001
TNF-α	36.00 (31–45)	145.50 (118–167)	< 0.001
GRP78	56.5 (23–90)	203 (103–260)	< 0.001
MAPK	48.5 (38–81)	185 (133–239)	< 0.001

## Discussion

Aging is a process that deeply affects individuals due to the health problems it causes, and societies due to the treatment costs of old age-related diseases and loss of labor^30,31^. In this context, the significance of experimental studies on the process of aging in animals is becoming increasingly apparent.

While testosterone is primarily known for its anti-inflammatory effects, estrogen has been shown to possess both anti-inflammatory and pro-inflammatory properties, as evidenced by its role in the pathogenesis of rheumatoid arthritis^32^. Furthermore, Carrol et al. demonstrated that beta-amyloid immunoreactivity was greater in female 3xTg-AD mice than in male 3xTg-AD mice^33^. In our study, female rats were used as the experimental subjects because the amyloid deposition profile, which is a consequence of the chronic inflammatory process in aged rats, was also investigated.

Aging is an inherent and unavoidable phenomenon that gives rise to histopathological and functional alterations in the kidneys, as well as in numerous other organs^34^. The study that compared the kidney morphology of six-month-old and 24-month-old mice revealed sclerotic glomeruli, thickening of the glomerular basement membranes, amyloid accumulation in the sclerotic glomeruli, and cast formations in the tubules of the kidneys in the 24-month-old mice^35^. Karakaya et al. observed glomerulosclerosis, thickening of tubular and glomerular basement membranes, tubular atrophy, loss of brush border, a decrease in the percentage of healthy-looking glomeruli, inflammatory cell infiltration in the interstitial space, vascular congestion and an increase in apoptotic cells in the tubules in aged rat kidneys that underwent pinealectomy^36^. Kremers and colleagues demonstrated that the number of sclerotic glomeruli in renal biopsy samples obtained from elderly individuals exhibited a statistically significant increase when compared to those from younger subjects^37^. The present findings are consistent with those reported in the aforementioned studies. Our findings indicated an increased percentage of sclerotic glomeruli in the elder rat group. However, although the mean GD/BD ratio in the elder rat group was lower than that in the young rat group, no statistically significant difference was found between the groups in terms of GD/BD ratios. This finding was explained by the coexistence of previously formed, shrunken, advanced-stage sclerotic glomeruli and newly formed sclerotic glomeruli that had expanded due to increased connective tissue in the mesangium in the elder group.

Oxidative stress, defined as the disruption of the oxidant-antioxidant balance in favor of oxidants, plays a role in aging through processes such as modification of histone proteins and DNA methylation^38,39^. The aging process and associated diseases lead to the production of reactive aldehydes such as malondialdehyde (MDA) through lipid peroxidation due to oxidative stress, which leads to protein carbonylation. Evidence has demonstrated that a disturbance in the redox balance between lipids and proteins in the elderly is associated with renal dysfunction. The high metabolic rate of the kidney renders it particularly vulnerable to oxidative damage, and the inflammation that results from reactive oxygen species (ROS) can perpetuate a detrimental cycle by exacerbating oxidative damage^40^. Protein oxidation has been shown to initiate the inflammatory cascade by causing the release of inflammatory signaling molecules, such as peroxiredoxin 2 (PRDX2). There is increasing evidence linking oxidative stress associated with diabetes, a common health problem in old age, to the production of proinflammatory cytokines such as TNF-α and IL-6, as well as the expression of inflammation-associated vascular cell adhesion molecule-1 (VCAM-1) and intercellular enzymes. It has been found that this process increases the expression of molecules such as Intercellular Adhesion Molecule 1 (ICAM-1) and nuclear factor κ-light-chain-enhancer of activated B cells (NF-κB), a transcription factor for proinflammatory cytokines. In addition, it has been shown that inflammasomes, which are an important part of the innate immune system, increase the production of IL-1β and IL-18 by activating caspase-1 in the kidney tissues of old rats^41–43^. Aging is defined as a process dominated by chronic, low-grade inflammation, and this inflammation is thought to play a role in the pathogenesis of age-related diseases^44^. Chan-ling et al. reported the presence of MHC class II + and ED1 + microglia cells and α β TCR + T lymphocytes in the retinas of old rats, suggesting low-grade inflammation. Notably, these cells were not detected in the retinas of young rats^45^. Dodiya and colleagues found that the inflammatory response to stress in mice resulted in the degradation of zonula occludens 1 (ZO1), claudin, and occludin 1 in the colonic mucosa, thereby exacerbating the symptoms of rotenone-induced Parkinson’s disease^46^. Jeong et al. observed increased levels of oxidative stress markers, such as MDA and myeloperoxidase (MPO), alongside elevated inflammatory markers, including TNF-α, IL-1β, IL-6, and IL-10, in the colonic tissue of aged rats compared to younger rats^47^. In the present study, in accordance with the extant literature, TNF-α and IL-6 levels were found to be elevated in the kidney tissues of aged rats in comparison with those of young rats. Furthermore, the study demonstrated that low-grade inflammation associated with the process of aging results in a reduction of occludin protein levels in kidney tissue.

Amyloid is thought to be a product of misfolding caused by errors in posttranslational modifications of proteins. Singh et al. have proposed that the alteration in protein conformation resulting from misfolding represents a primary event in the pathogenesis of Alzheimer’s disease. Additionally, they have suggested that Unfolded Protein Response (UPR) pathway mediators are elevated in the brain tissue of Alzheimer’s patients^48^. Protein folding in the ER is highly sensitive to intracellular and extracellular stimuli such as redox homeostasis, cytotoxicity, and inflammation. The UPR is a cellular mechanism that facilitates the adaptation of cells to ER stress by reducing protein translation and transcription, accelerating protein folding in the ER, and activating the ER-associated degradation (ERAD) process. GRP78, a pivotal molecule in the UPR process, functions as a regulatory molecule in the process of protein synthesis and folding. When necessary, the UPR is initiated through transmembrane proteins associated with ER stress. Additionally, it has been demonstrated that the UPR interacts with Tumor Necrosis Factor Receptor-Associated Factor 2 (TRAF2), and induces inflammation through the activation of c-Jun N-terminal kinase (JNK) and NF-κB signaling pathways, which are proteins linking oxidative stress and inflammation^49–51^. It has been established that free radicals and inflammatory cytokines can instigate ER stress, thereby creating a vicious circle between ROS, inflammation, and ER stress. The process of protein misfolding can directly result in the accumulation of ROS through an oxidative mechanism. The accumulation of ROS results in the disruption of calcium (Ca²⁺) channels located in the ER membrane, leading to the release of calcium from the ER into the cytosol. This then accumulates in the inner matrix of the mitochondria, ultimately disrupting the electron transport chain and leading to an increase in ROS production^52^. In fibrosarcoma cells, TNF-α has been shown to induce ER stress by stimulating UPR mediators^53^. In hepatocytes, cytokines such as TNF-α, IL-1β, and IL-6 can initiate the acute phase response via cyclic adenosine monophosphate (cAMP)-responsive element-binding protein H (CREBH) by inducing ER stress. Courties and colleagues demonstrated that TNF-α, IL-1, and IL-6 play a role in the accumulation of AA amyloid, which is synthesized from a plasma precursor produced by hepatocytes as an acute-phase reactant during chronic inflammation. In particular, IL-6 is associated with the severity and activity of amyloidosis^54,55^.

The primary target organs of systemic AA amyloidosis in animals have been reported to be the kidneys, skin, intestines, heart, spleen, and liver^56^. Similarly, the heart, kidneys, gastrointestinal tract, liver, peripheral and autonomic nervous systems, and skin are known to be affected in human systemic amyloidosis^57,58^.

In experimental animal models of amyloidosis, the inflammatory process is simulated using various methods, such as transgenic rodents with human familial Alzheimer’s disease mutations frequently introduced into their genomes^59^, subcutaneous soybean cream injection^60^, intraperitoneal lipopolysaccharide injection^61^, induction of chronic inflammation through methods like *Echinococcus multilocularis* inoculation^62^, gamma irradiation^63^, or parabiosis^64^. It is well established that, in cases of moderate ER stress, NF-κB activation is inhibited by CCAAT/enhancer-binding protein beta (C/EBPβ)^65^. Conversely, under severe ER stress, NF-κB activation and the inflammatory process are exacerbated through the c-Jun N-terminal kinase/protein kinase B (JNK/AKT) pathway^66^. Yu and colleagues demonstrated that Activator Protein 1 (AP-1) is the key transcription factor regulating the aging-associated inflammatory process in mouse kidney and liver tissues^67^. AP-1 is primarily activated by MAPK pathways. The three principal MAPK signaling pathway subfamilies, namely extracellular signal-regulated kinases (ERKs), p38, and JNK, are crucial for the activation of AP-1, thereby triggering AP-1-mediated cellular senescence and apoptosis^68^. While previous studies have shown that p44/42 MAPK is an important factor in the proliferation of age-associated rabbit vascular smooth muscle cells^69^ and that p44/42 MAPK activity is increased by IL-6 in IL-6-dependent plasma cell lines^70^, our study identified a novel association between p44/42 MAPK and age-associated low-grade inflammation.

The results indicate that low-grade inflammation mediated by NF-κB and MAPK, activators of AP-1 and JNK, is sufficient to induce histopathological and immunohistochemical changes in the kidney tissue of aged rats. However, this low-grade inflammation, triggered by these transcription factors, does not create a sufficiently pro-inflammatory microenvironment for amyloid deposition in organs that are primary targets of AA amyloid deposition, such as the kidney, heart, small intestine, and skin.

## Conclusions

The present study is the first to calculate the GD/BD ratio. This method may provide researchers with more reliable results in assessing morphometric changes in the glomerular tuft compared to measuring the diameter of the glomerulus or Bowman’s space alone. However, in the context of our study, in experimental models where the inflammatory stimulus is prolonged and ongoing—resulting in the coexistence of early and advanced-stage sclerotic glomeruli—the GD/BD ratio alone may be insufficient to reflect glomerulosclerosis.

A comprehensive review of the available literature did not reveal any previous studies directly linking low-grade inflammation with decreased occludin protein levels and increased GRP78 protein levels in aged rat renal tubular epithelial cells. We believe that our study will help fill this gap in the literature from this perspective as well.

The findings of our study, which investigated the relationship between NF-κB and MAPK-mediated proinflammatory microenvironment and amyloid accumulation in the kidneys of aged rats, revealed that aging-related low-grade inflammation induces histopathological changes in renal tissue but is not sufficient for amyloid accumulation in the primary target organs of systemic amyloidosis. The results suggest the existence of a threshold of inflammation and/or ER stress that, when exceeded, may lead to amyloid deposition or the involvement of additional transcription factors. The existence of specific transcription factors that cause amyloid deposition may also be the subject of future investigations.

### Limitations of the study

The results of our study are limited to rats. Also, due to budgetary constraints, it was not possible to evaluate oxidant-antioxidant markers, nor to perform confirmatory analyses such as Western blot and polymerase chain reaction (PCR).

## Supplementary Information

Below is the link to the electronic supplementary material.

Supplementary Material 1

Supplementary Material 2

Supplementary Material 3

Supplementary Material 4

## Data Availability

Data supporting the findings of this study are presented in tabular form in figshare (https://doi.org/10.6084/m9.figshare.28105127).

## Abbreviations

ER stress: endoplasmic reticulum stress
GRP78: Glucose Regulated Protein 78
SAA: Serum amyloid A
MDA: malondialdehyde
ROS: reactive oxygen species
PRDX2: peroxiredoxin 2
VCAM-1: vascular cell adhesion molecule-1
ICAM-1: Intercellular Adhesion Molecule 1
NF-B: nuclear factor-kappa B
ZO1: zonula occludens 1
MPO: myeloperoxidase
UPR: Unfolded Protein Response
TRAF 2: Tumour Necrosis Factor Receptor-associated Factor 2
JNK: c-Jun N-terminal kinase
CREBH: cyclic adenosine monophosphate (cAMP)-responsive element-binding protein H
C-EBPβ: CCAAT/enhancer-binding protein beta
JNK/AKT: c-Jun N-terminal kinases / protein kinase B
AP-1: Activator protein 1
MAPK: mitogen-activated protein kinase
ERKs: extracellular signal-regulated kinases
