# Supplementary material for: The effect of NF-kB and MAPK mediated Proinflammatory microenvironment on renal aging and amyloid deposition in elder rats
Source: Sci Rep. 2025 Aug 18;15:30188. doi: 10.1038/s41598-025-14559-y (PMC12361370; doi:10.1038/s41598-025-14559-y)
Supplement: Supplementary file 1 — Supplementary Material 1 [file 41598_2025_14559_MOESM1_ESM.docx]

**Supplementary Information Table.** Descriptive statistics and significance of the data obtained from the study

|  | Young (n=8) | Elder (n=8) | p* |
| --- | --- | --- | --- |
| GD/BD ratios | 0.857 (0.789-0.995) | 0.819 (0.73-0.865) | 0.127 |
| Percentage of sclerotıc glomerulus | 3.30 (1.32-9.15) | 10.10 (7.44-19.60) | **0.003** |

(GD: diameter of the glomerulus, BD: diameter of the Bowman capsule, p<0,05, * Mann Whitney U test.)

|  | Young (n=8) | Elder (n=8) | p* |
| --- | --- | --- | --- |
| Apoptotic cells | 0.60 (0.20-0.90) | 1.80 (1.50-2.20) | **< 0.001** |
| Cast formation | 0.05 (0.00-0.10) | 0.60 (0.10-1.70) | **0.001** |
| Tubular dilatation | 0.05 (0.00-0.20) | 0.40 (0.20-1.30) | **< 0.001** |
| Epithelial desquamation | 0.70 (0.60-1.00) | 2.00 (1.70-2.20) | **< 0.001** |
| Loss of brush border | 1.10 (0.90-1.50) | 2.6 (2.20-3.80) | **< 0.001** |

(p<0,05, * Mann Whitney U test.)
