# Supplementary figures and images for: The effect of NF-kB and MAPK mediated Proinflammatory microenvironment on renal aging and amyloid deposition in elder rats
Source: Sci Rep. 2025 Aug 18;15:30188. doi: 10.1038/s41598-025-14559-y (PMC12361370; doi:10.1038/s41598-025-14559-y)

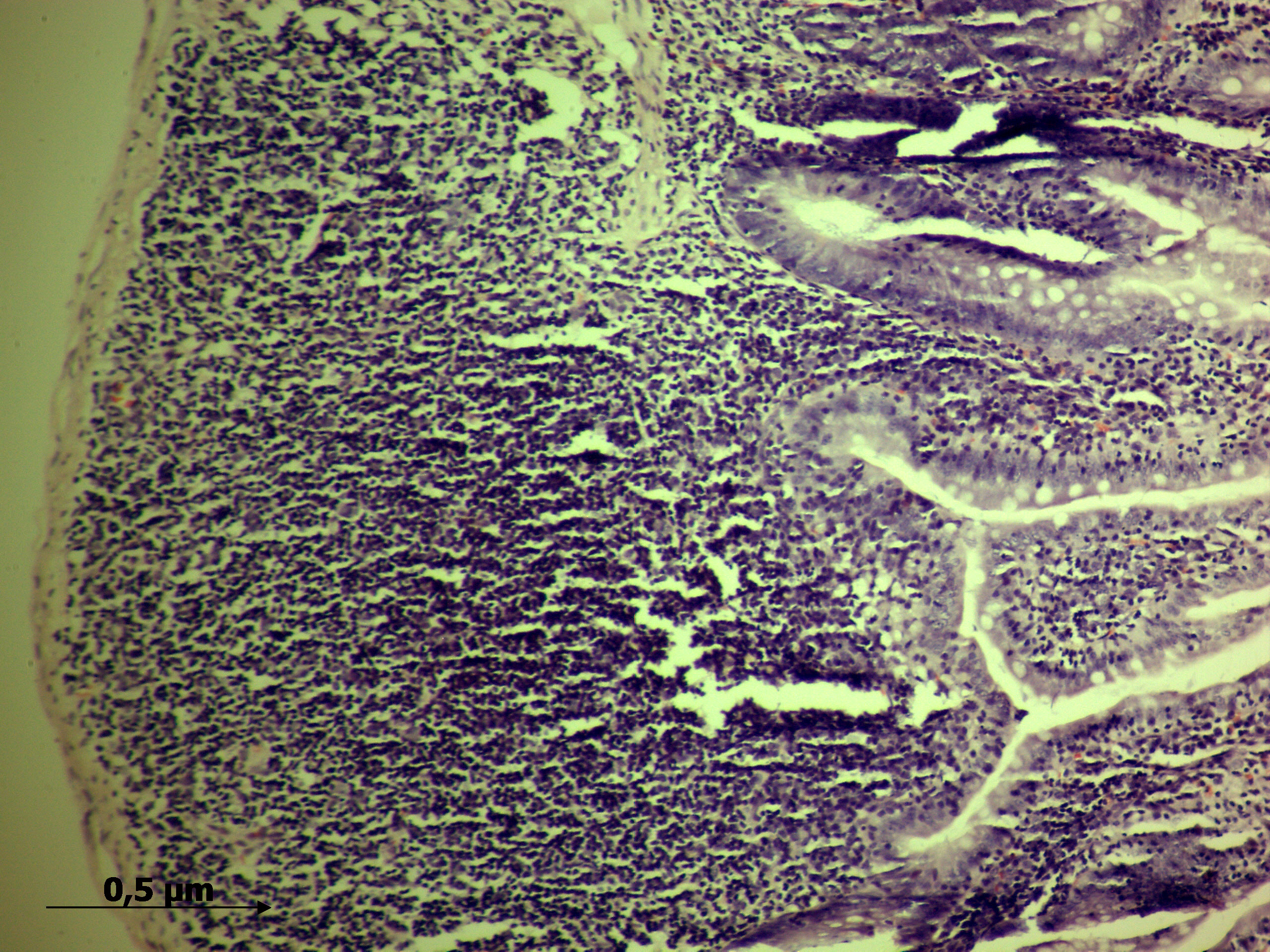

Supplement: Supplementary file 2 — Supplementary Material 2 [file 41598_2025_14559_MOESM2_ESM.jpg]

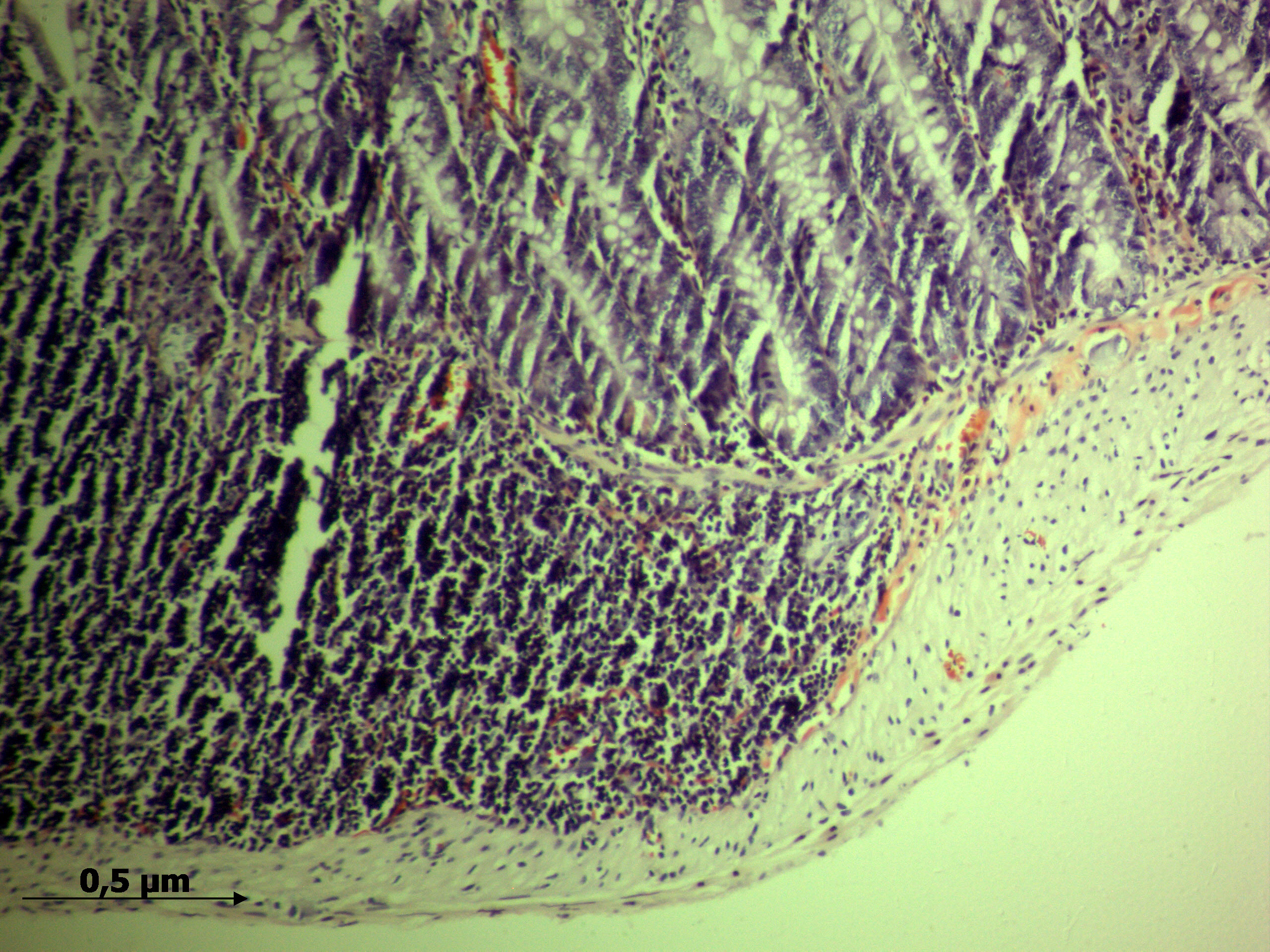

Supplement: Supplementary file 3 — Supplementary Material 3 [file 41598_2025_14559_MOESM3_ESM.jpg]
