# Supplementary material for: The effect of NF-kB and MAPK mediated Proinflammatory microenvironment on renal aging and amyloid deposition in elder rats
Source: Sci Rep. 2025 Aug 18;15:30188. doi: 10.1038/s41598-025-14559-y (PMC12361370; doi:10.1038/s41598-025-14559-y)
Supplement: Supplementary file 4 — Supplementary Material 4 [file 41598_2025_14559_MOESM4_ESM.docx]

**Supplementary materials**

1. **Supplementary information table.** This file provides supplementary analysis tables of the data obtained from the study, including descriptive statistics and significance levels.
2. **Supplementary Figure 1.** Images of ileum containing Peyer's patches in elder rats.
3. **Supplementary Figure 2.** Images of ileum containing Peyer's patches in young rats.
4. **Tissue processing.** Following fixation with 10% formalin, the kidney tissues were washed under running tap water and exposed to increasing alcohols (70, 90, 96 and 100%, respectively) for dehydration. After clarification with toluene, the tissues were embedded in liquid paraffin and blocked.
